# Loss of cIAP1 in Endothelial Cells Limits Metastatic Extravasation through Tumor-Derived Lymphotoxin Alpha

**DOI:** 10.3390/cancers13040599

**Published:** 2021-02-03

**Authors:** Lazaros Vasilikos, Kay Hänggi, Lisanne M. Spilgies, Samanta Kisele, Stefanie Rufli, W. Wei-Lynn Wong

**Affiliations:** Institute of Experimental Immunology, University of Zurich, CH-8057 Zurich, Switzerland; vasilikos@immunology.uzh.ch (L.V.); kfhb1985@gmail.com (K.H.); spilgies@immunology.uzh.ch (L.M.S.); kisele@immunology.uzh.ch (S.K.); rufli@immunology.uzh.ch (S.R.)

**Keywords:** cIAPs, Smac mimetics, tumor extravasation, endothelial barrier, permeability, lymphotoxin A

## Abstract

**Simple Summary:**

The ability of tumor cells to spread from one site to another within a patient is a critical factor in cancer survival. Tumor cell migration or metastasis is a complex process, which involves several stages. In this study, we specifically examine the stage in which the tumor cell must exit the blood stream. We find that the loss of cIAP1, a member of the inhibitors of apoptosis protein family (IAPs), alters the ability of tumor cells to exit the blood vessel or extravasate. Endothelial cell viability did not appear to be affected in the absence of cIAP1. Instead, we identified that the loss of cIAP1 hampers the response of endothelial cells to signals from the tumor cells to change shape and allow for migration through the endothelial barrier.

**Abstract:**

In this study, we determined whether Smac mimetics play a role in metastasis, specifically in circulation, tumor extravasation and growth in a metastatic site. Reports suggest inducing the degradation of IAPs through use of Smac mimetics, alters the ability of the tumor cell to metastasize. However, a role for the immune or stromal compartment in affecting the ability of tumor cells to metastasize upon loss of IAPs has not been defined. To address this open question, we utilized syngeneic tumor models in a late-stage model of metastasis. Loss of cIAP1 in the endothelial compartment, rather than depletion of cIAP2 or absence of cIAP1 in the hematopoietic compartment, caused reduction of tumor load in the lung. Our results underline the involvement of the endothelium in hindering tumor cell extravasation upon loss of cIAP1, in contrast to the immune compartment. Endothelial specific depletion of cIAP1 did not lead to cell death but resulted in an unresponsive endothelium barrier to permeability factors causing a decrease in tumor cell extravasation. Surprisingly, lymphotoxin alpha (LTA), and not TNF, secreted by the tumor cells, was critical for the extravasation. Using TCGA, we found high LTA mRNA expression correlated with decreased survival in kidney carcinoma and associated with advanced disease stage. Our data suggest that Smac mimetics, targeting cIAP1/2, reduce metastasis to the lung by inhibiting tumor cell extravasation.

## 1. Introduction

Elevated levels of cIAPs (cIAP1 and 2), in part due to genomic amplification, have been reported in a variety of human cancers such as hepatocellular carcinoma [1], lung cancer [2] and esophageal squamous cell carcinoma [3]. In cervical squamous cell carcinoma, elevated levels of cIAP1 are correlated with resistance to radiotherapy [4] and in colorectal and bladder cancer elevated levels of cIAPs are correlated with advanced stages of tumors and poor survival [3,4].

Because of the role of IAPs to inhibit cell death, pharmaceutical companies developed synthetic compounds to mimic the natural inhibitor of IAPs, Smac/DIABLO. The release of Smac from mitochondria results in autoubiquitination and degradation of cIAP1 and cIAP2 by binding to their BIR3 domain [5,6]. Smac also blocks XIAP through binding to the BIR2 and BIR3 domain, removing the inhibitory effect of XIAP on caspases [7]. Likewise, Smac mimetics cause the proteasomal degradation of cIAP1/2 and activation of the alternative NF-κB pathway. NF-κB drives TNF production and in the absence of cIAP1, TNFR1 activation leads to the formation of complex II and subsequent cell death [8,9,10,11].

In vitro cancer cell line screens show that Smac mimetics kill approximately 17% of tumor cell lines as a single agent [12]. However, since Smac mimetics cause innate immune cells to release TNF [13], tumor infiltrating lymphocytes are believed to aid in reducing tumor volume [14,15,16,17,18]. Additionally, in a B16-F10 subcutaneous tumor model, delivery of Smac mimetics in the tumor site disrupted neo-angiogenesis due to cell death which was rescued by the co-loss of TNFR1/2 [19]. Together, these results suggest that Smac mimetics affect the tumor microenvironment and may indirectly reduce the primary tumor burden.

The majority of patients die however, not due to the primary tumor but because of metastasis. For tumor cells to metastasize, the cells must enter the bloodstream, survive within the circulatory system and then extravasate at a secondary site [20]. Developmental studies show that the loss of cIAP1/2 results in hemorrhage and loss of endothelial cell integrity [21,22]. Tumor cell-induced endothelial cell death has been implicated as a mode of metastasis [23]. These data suggest that Smac mimetics would compromise endothelial cell integrity and enhance metastasis. Yet, current data suggests the use of Smac mimetics would reduce the ability of tumor cells to metastasize [24,25,26]. From this data, it is not clear whether Smac mimetics hinder tumor cell migration intrinsically or if the tumor microenvironment can actively reduce tumor cell metastasis.

In this study, we determine the role of Smac mimetics in the tumor microenvironment. The use of Smac mimetics is mimicked by the loss of cIAP1 or cIAP2 throughout the entire mouse without altering the expression level of IAPs in the tumor cell. The loss of cIAP1 but not cIAP2 in the endothelium obstructs tumor cell extravasation into the lung. Surprisingly, loss of cIAP1 does not enhance endothelium cell death during tumor extravasation and the results can be recapitulated using Smac mimetics (birinapant). Our data show that lymphotoxin alpha and not TNF derived from the tumor cells requires cIAP1 expression in the endothelium to induce tumor cell extravasation. These results suggest that application of Smac mimetics in the clinics might not only contribute to tumor killing by activating the immune system but also by preventing tumor metastasis of individual cancer types.

## 2. Results

### 2.1. Smac Mimetic Use Reduces Tumor Nodule Counts in the Lung Due to cIAP1 Loss and Not cIAP2

**Figure 1:**
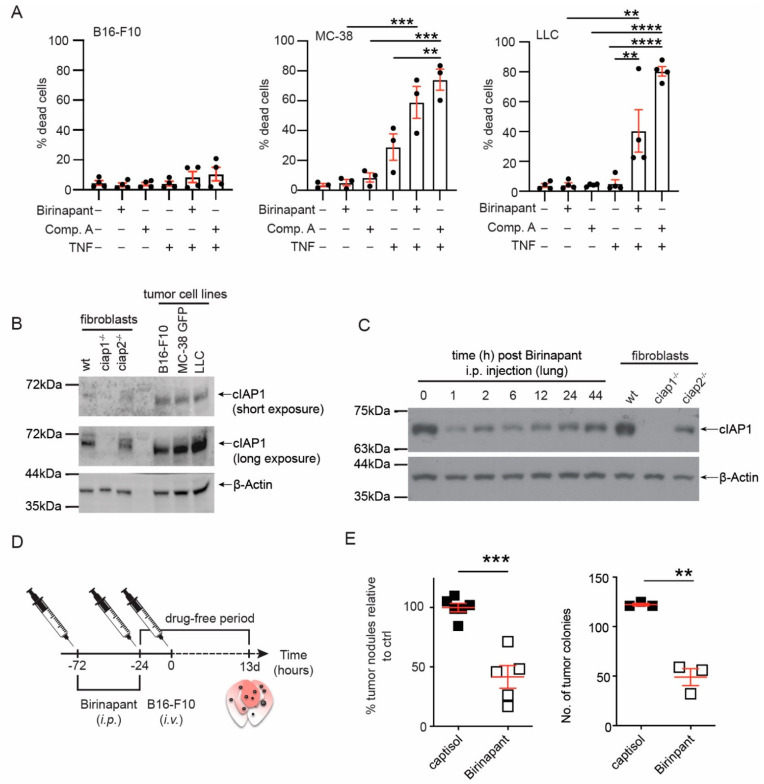
Smac mimetic, birinapant, reduces tumor nodules in the lung. (**A**) The cancer cell lines B16-F10, MC-38 GFP and LLC were treated with the Smac mimetics, birinapant (Bir, 500 nM) or Compound A (Comp. A, 500 nM), and/or mouse TNF (10 ng/mL). Comp. A is a bivalent Smac mimetic with affinity for XIAP, cIAP1 and cIAP2 [8]. Cell death was assessed by propidium iodide (B16-F10, LLC) or fixable viability dye eFLuor780 uptake using flow cytometry. (**B**) Levels of cIAP1 were assessed in the different cancer cell lines by Western blotting. A representative immunoblot is shown. (**C**) Wildtype (wt) mice were injected with birinapant (5 mg/kg, i.p.) and at the indicated time points, euthanized, lungs perfused, and total lung lysates were analyzed for cIAP1 by Western blotting. Shown is a representative immunoblot. The uncropped Western blots have been shown in Figure S6. (**D**) Experimental set up for Smac mimetic delivery and tumor cell injection. Wt mice were injected i.p. with birinapant (Bir, 5 mg/kg) or captisol (vehicle control) before the tumor challenge. Tumor cell nodules in the lung were counted 13 days post injection. (**E**) The number of tumor nodules counted in the lungs of mice treated with birinapant or vehicle control. To combine experiments, the number of tumor nodules for an individual mouse was normalized to the average number of tumor nodules counted in the lungs of mice treated with vehicle (captisol). Shown is a representative primary tumor nodule count. Each data point represents data obtained from an individual mouse. Data are presented as mean ± SEM with ** *p* < 0.01, *** *p* < 0.005 and **** *p* < 0.001 using one way ANOVA and multiple comparison test (**A**), two-tailed unpaired *t*-test (**E**).

To determine whether Smac mimetics would alter metastasis, we chose to inject tumor cells intravenously in the tail vein (B16-F10, melanoma; MC-38, colon carcinoma; LLC, Lewis lung carcinoma). In this model, tumor cells circulate to the lung, extravasate and form pulmonary tumor nodules [27]. Smac mimetics have been shown to cause tumor cell death in vivo because of exogenous TNF production from innate immune cells [14,16]. Therefore, we screened for a syngeneic tumor cell line, which did not die in response to Smac mimetics and TNF using viability stains and flow cytometry (Figure 1A). The gating strategy and representative FACS plots are shown (Figure S1A,B). In comparison to MC-38 or LLC tumor cells, B16-F10 cells only minimally increased in cell death in response to Smac mimetics and exogenous TNF stimulation. These data suggested B16-F10 cells in vivo would not die in response to birinapant and TNF produced by innate immune cells in the tumor microenvironment. The sensitivity to birinapant induced cell death with exogenous TNF was not linked to cIAP1 expression (Figure 1B). Next, we determined if cIAP1 loss occurred in the lung after injection of birinapant (5 mg/kg, i.p.). Loss of cIAP1 occurred within 1 h after birinapant injection and cIAP1 protein levels remained reduced up to 44 h (Figure 1C). In this model, tumor cells are thought to extravasate 6 h post tail vein injection [23]. We initially tried a schedule of birinapant injection 6 h prior to injection of tumor challenge (Figure S1C). No difference in the number of pulmonary tumor nodules was observed (Figure S1D,E). We then tried a different schedule of birinapant delivery, 72 and 24 h before the tumor challenge (Figure 1D). Mice that received birinapant had a 50% reduction in the number of tumor nodules counted on the lung (Figure 1E and Figure S1F). Because the number of tumor nodules varied between experiments, we normalized the number of tumors counted from an individual mouse to the average of the tumor counts of the vehicle control (captisol) treated mice and then combined independent experiments. These data supported that continuous use of birinapant would reduce rather than enhance the number of pulmonary tumor nodules. In addition, these data suggested the loss of cIAP1 in the tumor microenvironment may contribute to the inability of the tumor cells to metastasize in the presence of birinapant.

**Figure 2:**
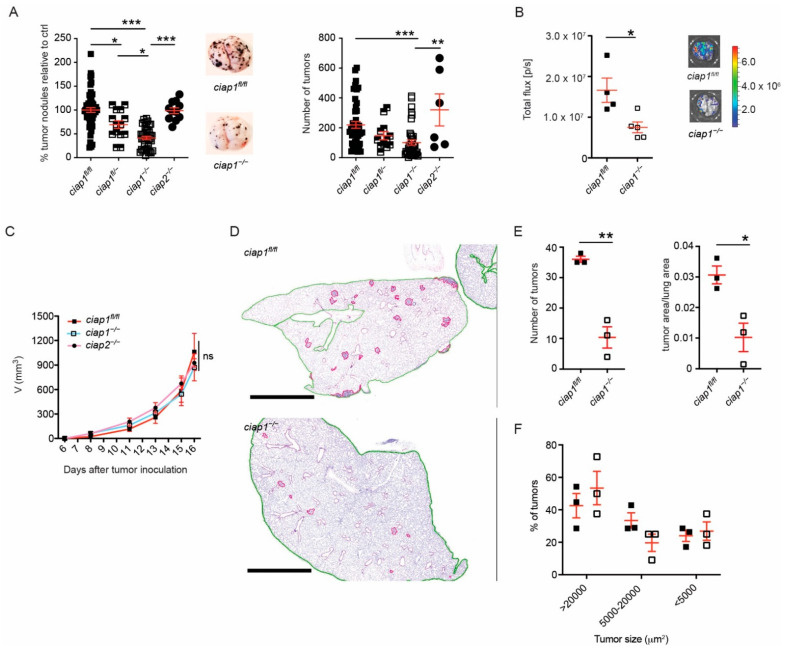
Loss of cIAP1 causes reduced tumor burden in a metastasis model. (**A**) Percentage of superficial B16-F10 tumor nodules in *ciap1^fl/fl^*, *ciap1^fl/-^*, *ciap1^−/−^* or *ciap2^−/−^* lungs normalized to the average number of tumor nodules identified in the Wt (*ciap1^fl/fl^*) control. Tumor cells were injected i.v. and nodules formed in the lung were counted 13 days later. Representative pictures of tumor-bearing lungs are shown. (**B**) B16-F10 cells expressing luciferase were injected i.v. and bioluminescence from the lungs was measured 13 days later utilizing the IVIS technology. Representative bioluminescence pictures are shown. (**C**) Subcutaneous tumor growth of B16-F10 cells. *ciap1^fl/fl^*, *ciap1*^−/−^ or *ciap2^−/−^* mice were injected subcutaneously with 100,000 tumor cells in the right flank and tumor growth was assessed using calipers (3–8 mice/group, representative experiment shown). (**D**) Representative H&E histology of lungs from *ciap1^fl/fl^* and *ciap1*^−/−^ post 13 days injection of B16-F10. Images were segmented for lung tissue (green outline) and tumor tissue (pink outline). Scale bar represents 1000 µm. (**E**) Quantification of tumor load by number and area. (**F**) Percentage of tumors by size identified in *ciap1^fl/fl^* and *ciap1^−/−^* lungs post 13 days injection of B16-F10 cells. Each data point represents data obtained from one animal. Data are presented as mean ± SEM. * *p* < 0.05; ** *p* < 0.01; *** *p* < 0.005, ns = not significant. One way ANOVA with multiple comparison (**A**), two way ANOVA with multiple correction (**C**) and two-tailed unpaired *t*-test (**B**,**E**)**.**

To clarify whether the loss of cIAP1 or cIAP2 played a dominant role in the tumor microenvironment in reducing the number of tumor nodules, we utilized *ciap1*^−/−^*ciap2^frt/frt^* (*ciap1*^−/−^) and *ciap1^fl/fl^ciap2*^−/−^ (*ciap2*^−/−^) mice to mimic the effect of Smac mimetics on the host alone. Approximately 50% fewer tumor nodules were identified in the lungs of *ciap1*^−/−^ compared to *ciap1^fl/fl^ciap2^frt/frt^* (*ciap1^fl/fl^*) mice, whereas *ciap1^fl/-^* mice showed an intermediate phenotype (Figure 2A). Tumor nodule counts were normalized to the average of tumor nodule counts obtained from lungs of *ciap1^fl/fl^* in independent experiments. Primary tumor nodule counts are also shown. However, the tumor load in *ciap2*^−/−^ mice was similar to wildtype mice, suggesting that the loss of cIAP1 was the reason for the reduction in tumor nodule numbers. To assess the tumor load in an unbiased manner, B16-F10 luciferase cells were utilized and a significant reduction in bioluminescence intensity was observed in *ciap1*^−/−^ mice, correlating with the tumor nodule counts (Figure 2B). To ensure tumor cells were not immunologically rejected in *ciap1*^−/−^ mice, we injected B16-F10 tumor cells subcutaneously. No difference in tumor growth kinetics among *ciap1^fl/fl^*, *ciap1*^−/−^ or *ciap2*^−/−^ mice was observed (Figure 2C). To determine if tumor growth is reduced in the lungs of *ciap1*^−/−^ mice resulting in undetectable tumors by eye, we assessed tumor burden by histological analysis. Lung and tumor tissue from mice injected with B16-F10 13 days post injection were segmented (Figure 2D and Figure S2A). We then compared the number of tumors and tumor tissue versus lung tissue areas in wildtype and *ciap1*^−/−^ lungs. *ciap1*^−/−^ mice had a consistent decrease in tumor burden (Figure 2E). We then determined whether the number of tumors of a particular size differed in lungs of *ciap1*^−/−^ mice compared to wildtype. Tumor nodules were divided based on area (>20,000, 5000–20,000, <5000 µm^2^) and each group was divided by the total number of tumors identified in the lung to give a percentage. The size of the detected tumors in *ciap1*^−/−^ lungs were similarly distributed in size as those in wildtype lungs (Figure 2F), suggesting the capacity of tumors to grow in *ciap1*^−/−^ lungs is possible. If growth was hindered in the lung of *ciap1*^−/−^ mice, we would expect a higher percentage of smaller sized tumors. However, this was not the case. To determine if cIAP1 loss in the tumor microenvironment could affect the number of tumor nodules formed in the lung by other syngeneic tumor lines, MC-38 GFP colon carcinoma or LLC cells were injected intravenously and 21 days post injection, the number of tumor nodules were counted. We observed a 75% reduction in MC-38 GFP tumor nodules in the lungs of *ciap1*^−/−^ compared to wildtype mice, while no reduction in tumor nodules was seen when LLC cells were injected (Figure S2B,C). These data suggest the loss of cIAP1 in the tumor microenvironment disfavors tumor cell accumulation in the lung or extravasation into the lung.

### 2.2. Loss of cIAP1 in the Hematopoietic Compartment Does Not Reduce Tumor Nodule Counts in the Lung

**Figure 3:**
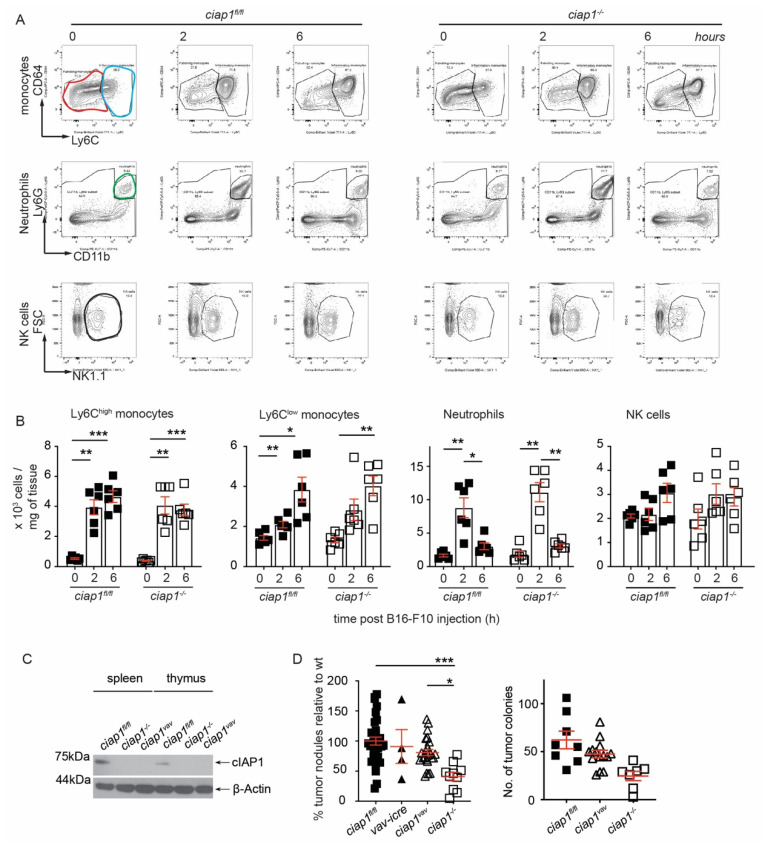
Loss of cIAP1 in the hematopoietic compartment does not alter the lung tumor load. (**A**) Representative FACS plots of lung immune cell infiltrates following B16-F10 tumor challenge by flow cytometry. Inflammatory monocytes (CD11b^high^MHCII^-^SiglecF^-^Ly6G^-^CD64^lo^Ly6C^high^, identified by blue gate), patrolling monocytes (CD11b^high^MHCII^-^SiglecF^-^Ly6G^-^CD64^lo^Ly6C^low^, identified by red gate), neutrophils (CD11b^+^Ly6G^+^, identified by green gate) and natural killer (NK) cells (CD3^-^NK1.1^+^, identified by black gate) were pre-gated on singlets, live and CD45^+^ cells (6 mice/group, performed twice). (**B**) Analysis of lung immune cell infiltrates following B16-F10 tumor challenge by flow cytometry shown in absolute numbers. (**C**) Splenocytes and thymocytes from *ciap1^vav^* mice were analyzed by Western blotting to evaluate the levels of cIAP1. Representative immunoblot is shown. (**D**) *ciap1^vav^* were challenged with B16-F10 tumor cells (i.v.) and nodules in the lung were counted 13 days later. Percentage of superficial B16-F10 tumor nodules in *ciap1^−/−^, ciap1^vav^*, Vav-icre and *ciap1^fl/fl^* normalized to the average number of tumor nodules identified in the Wt (*ciap1^fl/fl^*) in each independent experiment. A representative experiment showing primary tumor nodules is also shown. Each data point represents a mouse. Data are presented as mean ± SEM. * *p* < 0.05; ** *p* < 0.01; *** *p* < 0.001, two way ANOVA with multiple comparison test (**B**), one way ANOVA with Bonferroni’s test (**D**).

If the loss of cIAP1 in the tumor microenvironment affects the ability of tumor cells to reach the lung or to extravasate [28,29], we hypothesized a change in immune cell infiltrates may occur at the site of extravasation. Two important populations aid tumor cell extravasation, the inflammatory monocytes/macrophages (Ly6C^+^) and neutrophils (Ly6G^+^) [30,31] while natural killer cells (NK) are known to reduce the ability of tumor cells to circulate in the peripheral blood [20]. Surprisingly, these populations were similarly recruited to the lung at 2 h upon tumor challenge in both *ciap1^fl/fl^* and *ciap1^−/−^* mice (Figure 3A,B, gating strategies are shown in Figure S3A,B). Other innate and adaptive immune populations showed no major changes in kinetics in response to tumor challenge (gating strategies for immune populations, Figure S3C). No differences in factors involved in extracellular remodeling were observed in the absence of cIAP1 (Figure S3D), although macrophage metalloelastase-12 (MMP-12) was detected at higher amounts in unchallenged *ciap1^−/−^* lungs compared to wildtype. To determine whether the loss of cIAP1 in the hematopoietic cells affected the ability of tumor cells to reach the lung, we crossed *ciap1^fl/fl^ciap2^frt/frt^* with Vav-icre mice to deplete cIAP1 in the hematopoietic compartment (*ciap1^vav^*). cIAP1 deletion was confirmed by loss of protein detection in splenocytes and thymocytes (Figure 3C). Despite the loss of cIAP1 in the hematopoietic compartment, there was no reduction in the number of B16-F10 tumor colonies formed in the lungs of *ciap1^vav^* mice compared to *ciap1^fl/fl^* mice (Figure 3D). These data suggest that cIAP1 is not essential for the recruitment of immune cells early upon tumor challenge and that the immune system deficient in cIAP1 at steady state does not influence the ability of tumor cells to home to the lung.

### 2.3. Loss of cIAP1 in the Endothelial Compartment Reduces Tumor Cell Transmigration Independent of Cell Death

**Figure 4:**
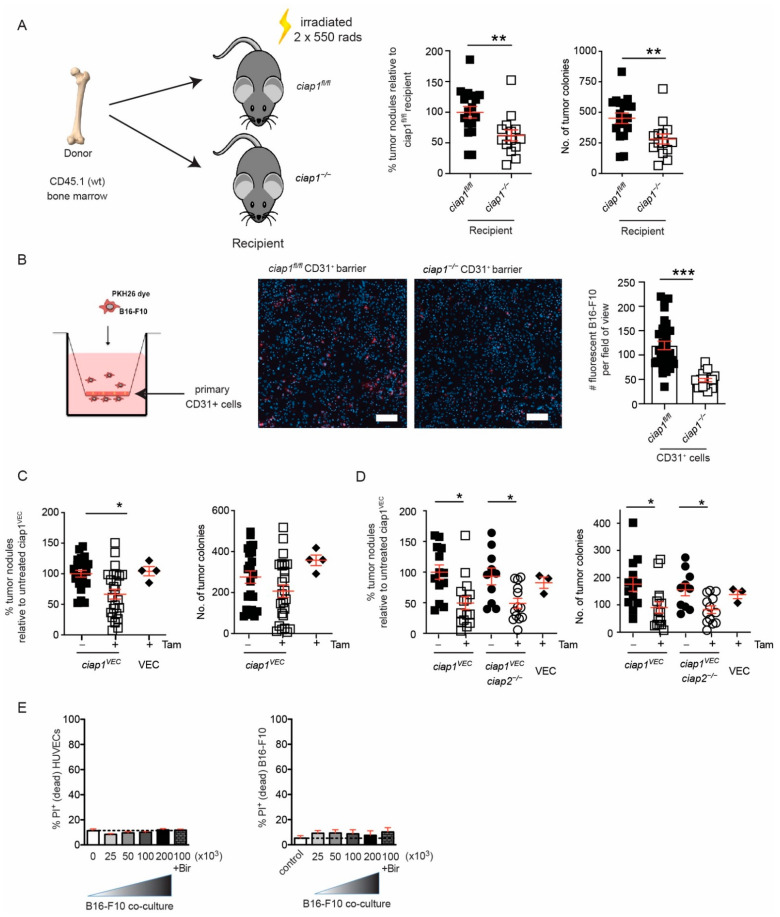
Transmigration of tumors is reduced due to loss of cIAP1 in endothelium barrier. (**A**) *ciap1^fl/fl^* and *ciap1^−/−^* mice were reconstituted with wildtype (CD45.1) bone marrow. Mice were challenged with B16-F10 tumors and lung nodules were counted 13 days later. Data shows the percentage of superficial B16-F10 tumor nodules from an individual mouse normalized to the average number of tumor nodules counted in the wildtype recipient control of an individual experiment. Primary tumor nodule count is also shown. (**B**) Transendothelial migration assays were performed with CellVue Plum-stained B16-F10 cells over a monolayer of primary isolated endothelial cells. Representative images of transwells demonstrating the amount of migrated CellVue Plum+ B16-F10 cells (red) surrounded by endothelial cells stained only with DAPI (blue). Scale bar is 100 µm. Each dot represents the number of manually counted CellVue Plum+ B16-F10 cells/field of view or image tile. *n* = 4–8 biological replicates. (**C**) *ciap1^VEC^* mice are challenged with B16-F10 tumor cells and tumor nodules are counted 13 days post injection. Percentage of superficial B16-F10 tumor nodules in *ciap1^VEC^, VEC* mice with and without tamoxifen (tam) oral gavage normalized to the average number of tumor nodules identified in *ciap1^VEC^* without tamoxifen. Primary tumor nodule count is shown alongside for comparison. (**D**) *ciap1^VEC^ciap2^−/−^* mice are challenged with B16-F10 tumor cells and tumor nodules are counted 13 days post injection. Percentage of superficial B16-F10 tumor nodules in *ciap1^VEC^ciap2^−/−^* and *VEC* mice with and without tamoxifen oral gavage (normalized to the average number of tumor nodules identified in ciap1^VEC^ without tamoxifen oral gavage). Primary tumor counts are presented alongside for comparison. (**E**) HUVEC monolayers were co-cultured with increasing numbers of CellVue Plum-stained B16-F10 cells, with or without birinapant, for 24 h. Cell death of HUVECs and tumor cells was assessed by propidium iodide (PI) uptake using flow cytometry (*n* = 3). Each data point represents a mouse. Data are presented as mean ± SEM. * *p* < 0.05; ** *p* < 0.01; *** *p* < 0.001. *t*-test (**A**,**B**). One way ANOVA with multiple comparison (**C**,**D**).

The transgene of Vav-icre mice was recently shown to disrupt expression of COMMD10 [27] and reported to drive loss of the floxed allele in endothelial cells. To determine if the loss of cIAP1 in the stromal compartment conferred reduction in lung tumor colonization, *ciap1^fl/fl^* and *ciap1^−/−^* mice were lethally irradiated and reconstituted with CD45.1 wildtype bone marrow. We observed a significant decrease in tumor nodules formed in the lungs of cIAP1 deficient recipients (Figure 4A). These results confirm that the loss of cIAP1 in the immune compartment does not play a role in reduced tumor burden in the lung and suggest tumor cells may not extravasate past a cIAP1 deficient endothelium barrier. To assess this possibility, primary endothelial cells from lungs of *ciap1^fl/fl^* and *ciap1^−/−^* mice were isolated (Figure S4A,B) for a transmigration assay. The loss of cIAP1 in endothelial cells effectively reduced the number of transmigrating B16-F10 cells by 60% (Figure 4B).

To specifically determine if the loss of cIAP1 in the endothelium was the critical compartment for the reduction of tumor colonies in the lungs, we crossed *ciap1^fl/fl^* mice with a tamoxifen inducible Cre line driven by the *Cdh5* (VE-cadherin) promoter [32] (*ciap1^VEC^*). Tamoxifen-fed *ciap1^VEC^* mice were injected with B16-F10 cells and showed reduced tumor colony numbers in the lung by approximately 50%, similar to *ciap1^−/−^* mice (Figure 4C). The efficiency of knocking out *ciap1* in the lung endothelium was confirmed by qPCR using primary isolated endothelial (CD31^+^) cells (75% reduction) (Figure S4C). Additionally, we crossed the *ciap1^vec^* to *ciap2^−/−^* mice to determine if the loss of cIAP1 resulted in a role for cIAP2 in reducing tumor cell extravasation. Combinatorial loss of cIAP1 and cIAP2 in the endothelium (*ciap1^VEC^ciap2^−/−^* mice) displayed a similar reduction in tumor load compared to *ciap1^VEC^ciap2^frt/frt^* mice, suggesting cIAP1 alone is responsible for obstructing tumor cell extravasation past the endothelium barrier (Figure 4D).

Apoptosis and necroptosis have been implicated in tumor transmigration [23,33] and the loss of cIAP1 has been shown to sensitize cells to apoptosis [8]. To determine whether tumor cells triggered the death of cIAP1 deficient endothelial cells, we co-cultured human umbilical vein endothelial cells (HUVECs) with various numbers of B16-F10 tumor cells and analyzed for cell death by flow cytometry. B16-F10 cells were membrane stained using CellVue Plum and the gating strategy for determining viability in the co-culture is shown (Figure S4D). Using birinapant in the media to simulate cIAP1 loss, we found no difference in the percentage of cell death in the HUVECs or the tumor cells, with or without birinapant (Figure 4E). Using a different tumor cell line, MC-38 GFP cells, we were able to confirm no difference in the percentage of cell death in the HUVECs or the tumor cells, with or without birinapant (Figure S4E). Together, these results suggest cIAP1 plays an important role in tumor cell extravasation through the endothelium barrier independent of cell death.

### 2.4. cIAP1 Aids in Tumor Extravasation by Promoting Permeability

**Figure 5:**
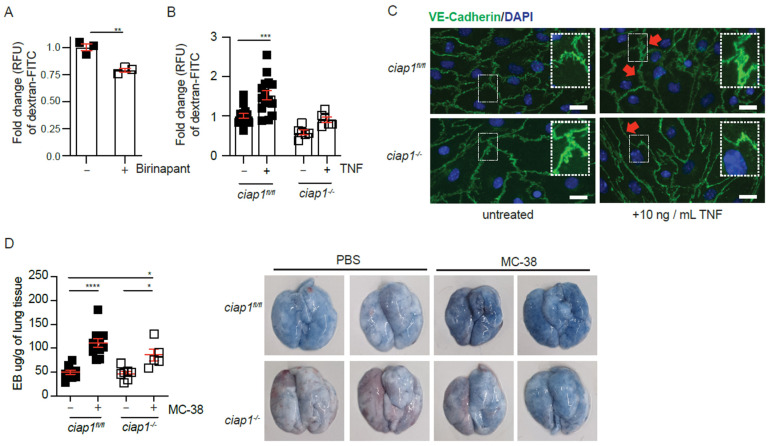
cIAP1 loss in the endothelium barrier affects permeability. (**A**) HUVEC monolayers were pre-treated with birinapant for 16 h before permeability to dextran-FITC was assessed (30 min). *n* = 3 transwells/condition (**B**) Primary isolated lung endothelial cell (EC) monolayers were stimulated with mouse TNF (TNF) for 18 h before permeability to dextran-FITC was assessed (30 min). Control cells were treated with DMSO or left untreated; *n* = 4 experiments with biological replicates. (**C**) Immunofluorescence staining of primary isolated lung ECs with DAPI (blue) and primary antibody against VE-Cadherin (green) treated with TNF for 18h. Scale bars: 20 μm. (**D**) Permeability assessment in vivo by Evans blue assay 6 h after injection of MC-38. Representative pictures of lungs after injection of Evans blue are shown. Each dot represents an individual mouse. Data are presented as mean ± SEM. * *p* < 0.05; ** *p* < 0.01; ****p < 0.0001. Two-tailed unpaired *t*-test (**A**), one way ANOVA with multiple comparison (**B**,**D**).

Our results suggest a non-apoptotic function of cIAP1 in endothelial cells aids in tumor cell extravasation. Previous data have shown inhibition of XIAP and cIAP1/2 in HUVECs reduces permeability in response to thrombin [34]. In agreement, birinapant reduced permeability of HUVECs in vitro at steady state, as measured by dextran-FITC permeability assay (Figure 5A). Similarly, the permeability of primary *ciap1^−/−^* endothelial cell barrier was reduced compared to *ciap1^fl/fl^* cells without stimulation. The addition of mouse TNF resulted in an increase in permeability in *ciap1^fl/fl^* but not in *ciap1^−/−^* endothelial cells (Figure 5B). In response to TNF, a known permeability factor, the junctions of VE-cadherin are disrupted in endothelial cells to promote permeability. In TNF treated *ciap1^−/−^* endothelial cells, VE-cadherin staining visually appeared tight without any breaks as assessed by eye. This is in contrast to TNF treated *ciap1^fl/fl^* cells, where VE-cadherin staining became less distinct (Figure 5C). These qualitative images of VE-cadherin staining suggested cIAP1 regulates the morphology and response of endothelial cells to permeability factors. Next, we assessed whether the absence of cIAP1 altered permeability in vivo in response to tumor challenge using the Evans blue assay. We did not detect a difference between basal level lung permeability between wildtype and *ciap1^−/−^* mice but in response to tumor challenge, lung permeability was decreased in the absence of cIAP1 (Figure 5D). Together, these data support a role for cIAP1 in tumor induced permeability.

### 2.5. Tumor Derived Lymphotoxin Alpha Is Responsible for Tumor Cell Extravasation

**Figure 6:**
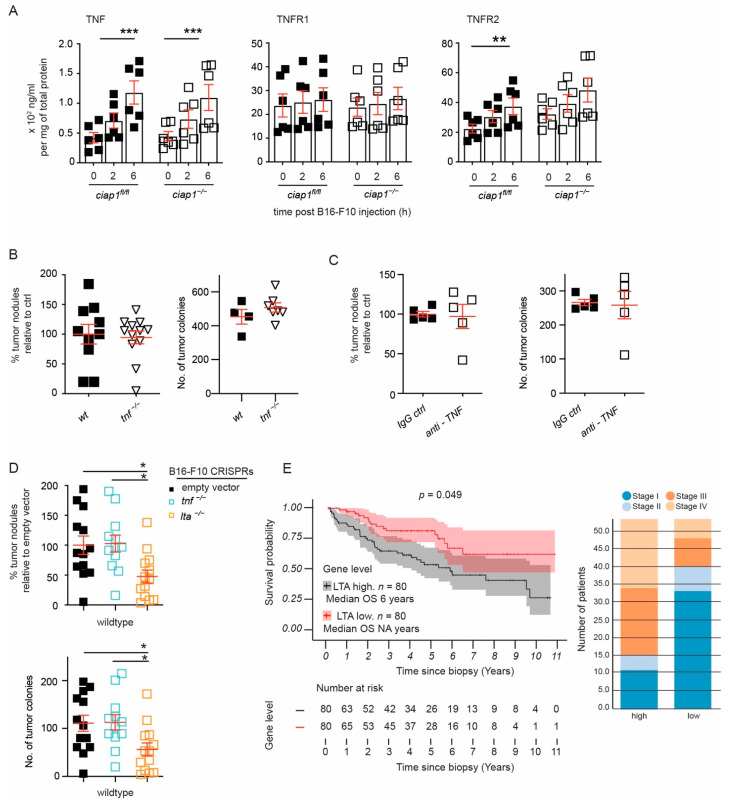
Loss of tumor derived Lymphotoxin A (LTA) reduces lung tumor nodules. (**A**) Levels of TNF and TNFRs assessed using multiplex analysis assay of total lung lysates (6 mice/group performed twice). (**B**,**C**) Wildtype, TNF deficient mice or wildtype mice pre-treated with anti-TNF (clone MP6-XT22) were injected with B16-F10 cells and tumor nodules in the lung were counted 12–13 days later. Percentage of superficial B16-F10 tumor nodules normalized to the average count of tumor nodules counted in the wildtype mice for an individual experiment. Primary tumor nodule counts are plotted for comparison. (**D**) LentiCRISPR-engineered B16-F10 cells against *tnf*, *lta* and control (empty vector) were injected *i.v.* into wildtype mice and nodules formed in the lung were counted 12 days later. Percentage of superficial B16-F10 tumor nodules normalized to the average number of tumor nodules identified in mice injected with B16-F10 control (empty vector). Primary tumor nodule counts are plotted for comparison. (**E**) Survival curve of high (>85th percentile) versus low (<15th percentile) LTA mRNA expression in kidney renal clear cell carcinoma from TCGA data and distribution of high versus low expression of LTA by disease stage. Data are presented as mean ± SEM. * *p* < 0.05; ** *p* < 0.01; *** *p* < 0.001. Two-way ANOVA with multiple comparison (**A**), two-tailed unpaired t-test (**B**,**C**), One way ANOVA with multiple comparison (**D**).

Since cIAP1 is well known to act downstream of the TNF/TNFRs pathway and TNF is a known cytokine involved in permeability, we examined whether TNF and TNFRs levels are influenced upon tumor challenge in the absence of cIAP1. Surprisingly, increasing levels of TNF could be detected in whole lung homogenates by luminex multiplex assay 2 h and 6 h post tumor challenge, suggesting that TNF might be important for the extravasation of the tumor cells. While the levels of TNFR1 did not change, the levels of TNFR2 increased after tumor challenge (Figure 6A).

TNF and LTA are the known ligands for TNFR1 and TNFR2. We found that the tumor load in *tnf^−/−^* mice was comparable to wildtype mice and injection of anti-TNF into the mice did not prevent tumor cells from extravasating and forming tumor nodules in the lung (Figure 6B,C and Figure S5A). Consequently, TNF or LTA in B16-F10 cells were targeted using CRISPR/Cas9. Polyclonal populations selected by drug resistance were sequenced for genetic loss. Similar to our previous data, loss of TNF in B16-F10 cells did not affect tumor burden. Surprisingly, the loss of LTA in B16-F10 caused a significant decrease in the tumor load (Figure 6D). These results suggest a mechanism in which B16-F10 tumor cell extravasation is mediated by the release of LTA and the subsequent activation of TNFRs.

To determine whether our finding was consistent in cancers which metastasize to the lung, we correlated LTA expression levels (mRNA) to cancer patient survival and disease stage using publicly available RNA expression data from The Cancer Genome Atlas (TCGA) [35]. Kidney, lung cancer, and melanoma are tumors known to metastasize to the lung. Among these tumor types, kidney renal clear cell carcinoma and papillary cell carcinoma displayed high expression of LTA mRNA (>85th quartile) that correlated with poor survival, while there was no correlation with TNF mRNA expression levels (Figure 6E, *p* < 0.05 and Figure S5B,C). We then segregated the high versus low expression by disease stage and found a great number of patients, categorized as disease stage 3 and 4, showing high expression of LTA. Surprisingly, in skin cutaneous melanoma and lung adenocarcinoma, the prognosis was the reverse, where high expression of LTA correlated with increased survival (Figure S5D,E). Taken together, these results suggest a metastatic strategy involving LTA.

## 3. Discussion

In vitro studies have shown that only a small fraction of tumor cell lines are susceptible to death upon Smac mimetics treatment as a single agent [12]. To determine whether there was an alternative application of Smac mimetics besides targeting the primary tumor, we examined the possibility of reducing metastasis. In our study, the B16-F10 murine melanoma cell line is not susceptible to Smac mimetics induced cell death even upon the combination of Smac mimetics and exogenous TNF. Using this as a tumor model to understand metastasis, we found that the absence of cIAP1 altered endothelial cell permeability and tumor cell extravasation. Surprisingly, despite the established role of immune cells in aiding metastasis, we found no alteration in the immune response during tumor cell extravasation when cIAP1 is lost. Instead, the loss of cIAP1 in the endothelium barrier reduced the response to permeability factors secreted by the tumor cell.

The complexity of understanding the metastatic cascade lies in the numerous interactions and cell types that are involved in this process. By focusing on the last stages of metastasis we were able to identify a novel role of Smac mimetics against tumor extravasation through host manipulation and without affecting the tumor cells. In agreement, Smac mimetics (JP1584) in combination with TRAIL antagonism have been used in an orthotopic syngeneic rat model of hepatic cholangiocarcinoma leading to a reduction in extrahepatic metastasis [36]. Moreover, Smac mimetic LCL161 was shown to suppress the growth of tumors in osteosarcoma mouse models via a TNF-induced cell death mechanism and consequently lung metastasis [25]. However, in these studies Smac mimetics target indiscriminately both the tumor cells and the tumor microenvironment and as a result the role of the host cannot be assessed during the various metastatic stages. Therefore, the promise of Smac mimetics as therapeutic agents might lie not only on targeting primary tumors but also being used as metastasis-reducing compounds in certain tumor types. Analysis of cIAP1 expression and protein location in the primary tumor may additionally indicate which tumors may benefit from Smac mimetic treatment to reduce metastasis [24]. Further investigation is required to determine if loss of cIAP1 will confer resistance to other sites of metastasis.

cIAPs have been implicated in endothelial cell integrity during development. The loss of cIAP1 in the zebrafish or cIAP1 and cIAP2 in the mouse leads to lethality due to hemorrhaging in the heart [21,22]. The loss of TNFR1 rescued heart development defects suggesting the initiation of an apoptotic program downstream of TNFR in endothelial cells. However, the loss of cIAP1 and cIAP2 in adult endothelial cells did not result in mortality of the mice. This suggests that past embryonic development, cIAP1 and cIAP2 play a different role in adult endothelial cells. Furthermore, induction of endothelial cell death in vivo by transmigrating tumor cells is not supported by our data. If loss of cIAP1/2 rendered endothelial cells sensitive to TNF or LTA induced apoptosis or necroptosis then an increase in tumor burden in the lung would be detected. Our results show the opposite, a dramatic decrease in tumor nodule numbers upon loss of cIAP1.

In vitro experiments using HUVEC and HMVECs (human microvascular endothelial cells) support that IAP antagonism with Smac mimetics (ABT or Smac085) prevents thrombin-induced hyperpermeability. Thrombin induced permeability required cIAP1 or XIAP to activate RhoA GTPase and subsequent actin re-arrangement [34]. We could not identify a role for RhoA GTPase using TNF stimulation and birinapant with HUVECs. However, in response to TNF, VE-Cadherin localization at the cell border did appear to weaken or become fuzzy in ciap1-/- endothelial cells. This suggests that cIAP1 could be involved in VE-cadherin phosphorylation via a Src-family tyrosine kinase, FYN as seen in human HMVECs [37].

Our data support that B16-F10 melanoma cells arrive at the extravasation site in the lung, where they express LTA to extravasate. LTA can activate both TNFR1 and TNFR2 on the endothelium to induce permeability/extravasation [38]. Activation of TNFR1 by soluble TNF is necessary to induce endothelial permeability in vitro and in vivo. Interestingly, TNFR2 may play a role in permeability as well, dependent on the organ [39]. Whether tumor-derived LTA preferentially utilizes TNFR1 or TNFR2 to promote permeability in the lung vessels will require further study. This may be one of the mechanisms utilized by tumor cells to induce permeability. Primary tumors known to metastasize to the lung include breast cancer, melanoma, and renal carcinoma. Not all of these cancers showed a correlation with survival and expression of TNF/LTA with the exception of renal carcinoma. Taken together, these results suggest further work to explore the relevance of high LTA in renal carcinoma in mouse models and in a de novo human sample set should be explored.

Besides birinapant (NCT00993239, NCT01681368), there are several other Smac mimetic compounds that have reached clinical trials. Among them are LCL161 (NCT01098838), GDC-0152 (NCT00977067, NCT01226277, NCT01908413) and Debio1143 (NCT01078649) and the results of these clinical trials has been recently summarized [40]. These clinical trials show that in a range of different tumor types, Smac mimetic compounds are unlikely to be effective as a single agent in reducing the primary tumor load. However, Smac mimetics in combination with current chemotherapeutics show promise, increasing survival rates to 18 months for advanced squamous cell carcinoma of the head and neck (Debio1143 combined with high-dose cisplatin chemoradiotherapy) [41]. Cytokine release syndrome may be a limiting adverse factor, potentially driven by the loss of IAPs in innate immune cells [13] and will still need to be monitored. Biomarkers identifying which tumor types are likely to show success with Smac mimetics are limited although a recent publication suggests in triple negative breast cancer tumors, high mRNA expression of TNF, RIPK1 and STX37 are indicative of response [42]. Whether these identified markers will translate to tumors inhibited in metastasis by Smac mimetics is unknown. No clinical trial has been designed to specifically examine the potential of Smac mimetics to reduce metastasis, however, without identifying which tumor types or potential markers of sensitivity, constructing such a trial will be difficult. Yet, in searching for therapies to prolong survival, reducing metastasis may be one essential aspect worth investigating.

## 4. Materials and Methods

### 4.1. Animal Work

Animals were maintained in specific pathogen free (SPF) and optimized hygiene conditions (OHB), and experiments were approved by Zurich Cantonal Veterinary Committee in accordance with the guidelines of the Swiss Animal Protection Law (License 119/2012 and 186/2015). *ciap1^fl/fl^ciap2^frt/frt^* (*ciap1^fl/fl^*), *ciap1^−/−^ciap2^frt/frt^* (*ciap1^−/−^*), *ciap1^fl/fl^ciap2^−/−^* (*ciap2^−/−^*) mice were obtained from Dr. John Silke (WEHI, Melbourne, Australia); Vav1-icre (vav) from Jackson Labs; Tg(Cdh5-cre/ERT2)1Rha (VEC) from Prof. Dr. Ralf Adams. Age and sex matched mice (6–12 weeks) were injected with B16-F10 (10^6^ cells/mL), MC-38 (1.5 × 10^6^ cells/mL) or Lewis lung carcinoma (LLC) (1.5 × 10^6^ cells/mL) intravenously in 200 μL PBS or subcutaneous in 100 μL PBS. Mice were sacrificed by anesthesia, lungs perfused and blinded before further processing (nodule counting, flow cytometry analysis). Once nodule counting or flow cytometry analysis is complete, the samples are unblinded and graphed. Subcutaneous tumors were injected only in female mice (6–12 weeks old). For bone marrow chimeric experiments, mice were irradiated two times at 5.5Gy (11Gy total dose), 15 h apart, injected with CD45.1 wildtype donor bone marrow, supplied with antibiotics for the first three weeks (neomycin into drinking water at 1mg/mL) and assessed for reconstitution efficiency at 6 weeks. Only mice with reconstitution efficiency >95% were used for further experiments. For the analysis of tumor nodule counts, the number of tumor nodules counted for an individual mouse were normalized for comparison among independent experiments using the average of the designated control in the experimental group. For example, in a comparison of the number of tumor nodules formed in *ciap1^fl/fl^* and *ciap1^−/−^* mice, the *ciap1^fl/fl^* mice would be considered the control. The lung of each mouse in the experiment would be individually counted for tumor nodules. The average number of tumor nodule counts in the *ciap1^fl/fl^* mice would be the comparator. For luciferase imaging, mice were injected with D-luciferin (150 mg/kg; Pierce, Germany), euthanized and the lungs were imaged using a Xenogen IVIS 100 (Caliper Life Sciences, Hopkinton, MA, USA) imaging system. Luminescence was recorded and the photon flux was analyzed using the Living Image 2.5 software (Caliper Life Sciences).

### 4.2. Cell Culture and Viability Assays

Tumor cells were cultured at maximum 80% confluency in high glucose (4.5 g/L) Dulbecco’s modified Eagle’s medium (DMEM) (MC-38 GFP, LLC) or RPMI 1640 (Gibco, ThemoFisher Scientific, Zug, Switzerland) (B16-F10) supplemented with 10% fetal bovine serum (FBS; Gibco, ThemoFisher Scientific) and penicillin/streptomycin/glutamine (PSG, Gibco, ThemoFisher Scientific). For B16-F10 cells, media was switched to high glucose DMEM 48 h prior to injection to stimulate melanin production. Primary lung ECs and HUVECs (C-12200, Promocell, Heidelberg, Germany) were cultured on gelatin pre-treated plates (0.1% gelatin) in EBM-2 media supplemented with the EGM-2 SingleQuot Kit according to the manufacturer’s recommendation (Lonza, Basel, Switzerland) or the equivalent Endopan 3 medium (PAN Biotech, Aidenbach, Germany). Analysis of viability was assessed by plating endothelial cells and/or tumor cells, treating with compound or ligand, harvesting cells and staining using viability dyes such as propidium iodide (1 μg/mL), eFluor780 viability stain or Amcyan (ebioscience, Vienna, Austria) as per manufacturer’s instructions.

### 4.3. Primary Lung EC Isolation and VE-Cadherin Staining

Lungs were perfused with PBS and HBSS+Collagenase IV (1 mg/mL, Sigma-Aldrich, Buchs, Switzerland) and minced before incubation with HBSS+Collagenase IV for 45 min at 37 °C and a cell suspension was obtained. Mouse lung cell suspension was incubated with anti-CD31 antibody (clone 390, London, UK, BioLegend) for 30 min in PBS+0.5% BSA at 1:25 dilution in order to isolate lung ECs, followed by incubation with anti-rat IgG magnetic particles and sorted on LS columns according to the manufacturer’s recommendations (Miltenyi, Bergisch Gladbach, Germany). Isolated cells were allowed to settle on coverslips overnight. If required, cells were treated with mouse TNF for various time points and fixed with 2% paraformaldehyde, permeabilized with 0.2% Triton X100 in PBS and stained with anti VE-Cadherin (clone 11D4.1, BD Bioscience, Allschwil, Switzerland). Coverslips were mounted on glass slides using SlowFade Gold with DAPI (ThermoFisher Scientific). Fluorescent images were taken with an Olympus IX81 inverted fluorescent microscope using 10× objective, 0.35 NA or 40× objective power, 0.6 NA. VE-cadherin thickness was not assessed by software but was observed manually.

### 4.4. Transendothelial Migration Assay

Only EC populations of >90% purity (as assessed by CD31^+^) were used. Briefly, 3 × 10^4^ primary ECs were seeded 3–4 days after isolation on a gelatin coated insert (Corning, Netherlands, 8 μm pore size) in EBM-2 (Lonza) or Endopan3 (Pan Biotech). Two days after seeding, cell culture media was changed to RPMI 1640 (Gibco, ThermoFisher) in the bottom well (3% FBS) and insert (1% FBS) and 2 × 10^4^ CellVue Plum^+^ (Polysciences, Hirschberg an der Bergstrasse, Germany) or PKH26^+^ (Sigma-Aldrich) membrane stained B16-F10 tumor cells were added to the insert. After 20 h, the remaining cells inside the insert were removed gently with a cotton swab, the membrane was fixed in 2% PFA and outer membrane side was mounted on glass slides using SlowFade Gold with DAPI (ThermoFisher). Fluorescent images were taken with a ZEISS AXIO Scan Z.1 (10× objective power, 0.45 NA with a 16-bit color camera; Hamamatsu Orca Flash 4.0 monochrome camera, 2048 × 2048 pixels, 4 MP, pixel size 6.5 µm) at the Centre for Microscopy and Image Analysis (ZMB), Switzerland. Images were exported as 6 × 6 tiles and five image tiles in the middle of the membrane were counted per image. These tiles were blinded, given to a second researcher for manual counting of cells (identified by DAPI nuclear stain) with CellVue Plum^+^ or PKH26^+^ staining. No digital image analysis was performed. Samples were then unblinded for graphing. Individual counts/tile are shown.

### 4.5. Vascular Permeability Assay

Evans Blue assay: Permeability of the lung microvasculature was determined by Evans blue dye similar to Wolf et al. [43]. Mice were injected with MC-38 GFP tumor cells (1.5 × 10^6^ cells/mL) then 6 h later, the mice were injected with Evans blue at 20 mg/kg (2% solution in PBS). After 30 min, mice were euthanized, lungs were perfused, dissected, photographed, and homogenized. Evans blue was extracted by incubation with formamide at 60 °C for 16 h. Evans blue concentration was measured by absorbance at 620 nm and corrected for erythrocyte contamination using 740 nm.

Dextran-FITC permeability assay: 3 × 10^4^ HUVEC or primary ECs were seeded 3–4 days after isolation on a gelatin-coated insert (Corning, 8 μm pore size) in EBM-2 (Lonza) or Endopan3 (Pan Biotech) and grown to a confluent monolayer for 3 days. The medium was gradually replaced to ECGS medium (low glucose DMEM + 10% FBS + PSG + ascorbic acid (EBM-2 supplement, Lonza) + heparin (EBM-2 supplement, Lonza) + 30μg/mL endothelial growth supplement from bovine pituitary (ECGS, Sigma-Aldrich). Cells were then pre-treated with mouse TNF or birinapant for 18 h before dextran-FITC (70kDa, Sigma-Aldrich) was added into the insert at final concentration of 1 mg/mL. Supernatant from the bottom well was collected 30 min after addition of dextran-FITC and fluorescence intensity was measured by TECAN Infinite 200 Pro multireader (Switzerland) using λEx = 490 nm and λEm = 520 nm.

### 4.6. Multiplex Assay

Total lung lysates were prepared according to the manufacturer’s instructions. A customized mouse Magnetic Luminex assay kit was used (Bio-Techne, Minneapolis, MN, USA). The measurements were performed on a Bio-Rad BioPlex 200 system (Bio-Rad Laboratories, Basel, Switzerland) and data were analyzed with the Bio-Plex software 6.0.

### 4.7. Antibodies and Viability Dyes

The following antibodies/viability dyes were used: CD11b-PE/Cy7 (M1/70), CD45-BV605 (30F-11), Ly6C-BV711 (HK1.4), CD11c-APC/Cy7 (N418), Ly6G-PerCP/Cy5.5 (1A8), MHCII-AF700 (M5/114.15.2), CD64-APC (X54-5/7.1), CD3-BV785 (17A2), CD4-APC (GK1.5), CD8-PerCP (YTS156.7.7), NK1.1-BV650 (PK136) from Biolegend; CD45-AF700 (30F-11), CD24-PE (M1/69), CD103-FITC (2E7), fixable viability dye eFluor506 and eFluor780 from eBioscience; CD144 (VE-cadherin, 11D4.1), SiglecF-BV421 (E50-2440), CD11b-BV605 (M1/70) from BD Biosciences; donkey anti-rat IgG (H+L)-AF488 from Jackson; propidium iodide (PI), beta-actin (AC-15) (Sigma-Aldrich); anti-cIAP1 (1E1-1-10; Enzo Life Sciences or HAP002317 (Atlas Antibodies) using wildtype, ciap1^−/−^, ciap2^−/−^ fibroblasts as controls for antibodies; goat anti-rat IgG (H+L)-HRP from ENZO Life sciences (ADI-SAB-220-0500), donkey anti-mouse and anti-rabbit IgG (H+L)-HRP from SouthernBiotech (Birmingham, AL, USA).

### 4.8. Ligands and Inhibitors

Mouse and human TNF (10 ng/mL, purified from mammalian expression system pcDNA5 FRT fc hs TNFa and pCR3 fc ps mm TNF; kind gift from J. Silke), birinapant (500 nM, Chemietek, Indianapolis, IN, USA), Compound A (12911, 500 nM, Tetralogics, Malvern, PA, USA).

### 4.9. Compounds Used In Vivo

Mice received an intraperitoneal injection of 200 μg of IgG control or anti-TNF antibodies (clone MP6-XT22, Biolegend) in 100 μL PBS 2.5 h before the tumor challenge (10^5^ B16-F10 cells in 100 μL of PBS). Birinapant was dissolved in 12.5% captisol (5 mg/mL, Captisol, KS, USA) and used for intraperitoneal injections at 5 mg/kg after 1:10 dilution in PBS. Tamoxifen (Monmouth Junction, NJ, USA, MedChemExpress) was dissolved in corn oil (Sigma-Aldrich) at 20 mg/mL. Young mice (5 weeks old) were fed for 3 consecutive days by oral gavage (150 mg/kg).

### 4.10. Western Blotting

Organs (lung, spleen, thymus) were lysed using DISC lysis buffer (20 mM Tris-HCl pH 7.5, 150 mM NaCl, 10% (*v*/*v*) glycerol, 1% (*v*/*v*) NP-40, 2 mM EDTA, 5 mM EGTA, 30mM NaF, 40 mM b-glycerophosphate pH 7.2, 10 mM sodium pyrophosphate, 2 mM activated sodium orthovanadate, protease inhibitors). The insoluble fraction of the lysate was pelleted by centrifugation and removed. Lysates were boiled and run on 4–20% Bis-Tris Gel NuPAGE using MOPS buffer (ThermoFisher Scientific). Proteins were then transferred onto PVDF-membrane (0.2 µm, ThermoFisher Scientific) using the Trans-Blot^®^ Turbo™ Transfer System (Bio Rad), according to the manufacturer’s instruction. After blocking with PBST containing 5% (*w*/*v*) skim milk, membranes were incubated with the indicated primary antibody in PBST containing 5% skim milk overnight at 4 °C, followed by incubation with the respective secondary antibody. Protein level expression was visualized on a film (Kodak, Rochester, NY, USA) using chemiluminescence (WesternBright ECL, Advansta, San Jose, CA, USA) or FusionFX7 (Witec AG, Heitersheim, Germany) with an aperture of 0.84 and binning 1 × 1.

### 4.11. qPCR

RNA from primary isolated lung ECs was extracted using Genezol (Geneaid, New Taipei City, Taiwan) according to the manufacturer’s instructions. cDNA was produced using MultiScribe™ Reverse Transcriptase and SYBR Green qPCR master mix (Thermo Fisher Scientific) was used for running the qPCR. Melting curves showed that single products were formed. The following primers were used: mouse *ciap1* (forward, 5-AATGGTTTCCAAGGTGTGAG; reverse, 5-GGACAACAGCTGCTCAAG); mouse *B2M* (forward, 5-TGGTGCTTGTCTCACTGACC; reverse, 5-CCGTTCTTCAGCATTTGGAT). Relative standard curve analysis was performed using the housekeeping gene, *B2M,* and ECs from non-tamoxifen fed mouse were used as a calibrator for fold-change.

### 4.12. Lentiviral CRISPR/Cas9 Constructs

The LentiCRISPRv2 one vector system was utilized to target the *tnf* and *lta* genomic loci in B16-F10 melanoma cells. The following oligos were used in order to clone the desired target sequence into the vector: mouse *lta* CRISPR (forward, 5-CACCGGGGTGCCAAGCACCCTCAAG, reverse, 5-AAACCTTGAGGGTGCTTGGCACCCC), mouse *tnf* CRISPR (forward, 5-CACCGAGAAAGCATGATCCGCGACG; reverse, 5-AAACCGTCGCGGATCATGCTTTCTC). Oligos were designed using the Benchling CRISPR gRNA Design tool (http://www.benchling.com). For cloning, viral production, tumor cell viral transduction, and selection, we followed the recommended guidelines [35,36].

### 4.13. mRNA Expression Level Analysis and Survival Curves

Using the platform by Cheng et al., RNAseq [35], we examined the mRNA expression level in the TCGA datasets. Based on the mRNA expression level for a given tumor type, patient survival was plotted against tumors with the highest (top 15%) and lowest (bottom 15%) mRNA expression.

### 4.14. Statistical Analysis

All data is presented in mean ± SEM. Figures were prepared in Illustrator CC 2015 (Adobe, San jose, CA, US) and Prism 5 (GraphPad Software). Significance between genotypes and treatments was assessed by Student-*t* test, one way or two way ANOVA with * *p* < 0.05, ** *p* < 0.01, *** *p* < 0.001, ns = not significant using Prism 9.

### 4.15. Histology and Tumor Mass Analysis

Lungs were perfused with saline and fixed using 4% paraformaldehyde overnight. The lungs were dehydrated and embedded in paraffin blocks. 5 µm histology cuts were done using a Zeiss Hyrax M55 rotary microtome (Switzerland). Staining was performed using Hematoxylin and Eosin (VWR). Imaging was performed on the Zeiss Axio Scan Z.1 slide scanner (20× objective power, 0.8 NA and a 8 bit Hitachi HV-F202FCL color camera (1600 × 1200 pixels, 2 MP, pixel size 4.4 μm). CZI files were exported as a TIFF file in order for the tumor tissue to be annotated manually by using ImageScope 12.4.3.5008 software (LEICA, Wetzlar, Germany). Lung sections were annotated for tumor tissue and total lung tissue, excluding large bronchi and other non-lung tissues.

## 5. Conclusions

Our study shows that the loss of cIAP1 but not cIAP2 alters the ability of endothelial cells to respond to permeability factors. This results in a decreased number of tumor cells extravasating through the endothelial barrier in a tumor-derived lymphotoxin alpha manner. Taken together, these findings identify that Smac mimetics can alter the stroma compartment of the tumor microenvironment, reducing metastasis to the lung.

## Data Availability

Data is available upon request. Immune infiltrate flow cytometric data has been deposited at https://flowrepository.org/.

## Supplementary Materials

The following are available online at https://www.mdpi.com/2072-6694/13/4/599/s1, Figure S1: Cell viability analysis of different cancer cell lines upon Smac mimetics treatment, Figure S2: Tumor cells are preferentially reduced in lungs of *ciap1^-/-^* mice but not due to immune rejection, Figure S3: Loss of cIAP1 in immune infiltrates do not reduce tumor nodules in the lung, Figure S4: Isolation of primary endothelial cells (ECs), verification of cIAP1 loss by tamoxifen driven cre and analysis of cell death of MC-38 cells co-cultured with HUVEC monolayers, Figure S5: Correlation between survival of cancer patients and expression levels of LTA and TNF, Figure S6: The uncropped Western blots.
