# Supplementary material for: Loss of cIAP1 in Endothelial Cells Limits Metastatic Extravasation through Tumor-Derived Lymphotoxin Alpha
Source: Cancers (Basel). 2021 Feb 3;13(4):599. doi: 10.3390/cancers13040599 (PMC7913358; doi:10.3390/cancers13040599)
Supplement: Supplementary file 1 [file cancers-13-00599-s001.pdf]

cancers

Loss of cIAP1 in Endothelial Cells Limits Metastatic Extravasation through Tumor-Derived Lymphotoxin Alpha

Lazaros Vasilikos, Kay Hänggi, Lisanne M. Spilgies, Samanta Kisele, Stefanie Rufli and W. Wei-Lynn Wong

Citation: Vasilikos, L.; Hänggi, K.; Spilgies, L.M.; Kisele, S.; Rufli, S.; Wong, W. W.-L. Loss of cIAP1 in Endothelial Cells Limits Metastatic Extravasation through Tumor-Derived Lymphotoxin Alpha. *Cancers* **2021**, *13*, 599. https://doi.org/10.3390/cancers13040599

Academic Editor: Lisa M. Sedger, Heinrich Korner

Received: 24 December 2020
Accepted: 29 January 2021
Published: 3 February 2021

Publisher's Note: MDPI stays neutral with regard to jurisdictional claims in published maps and institutional affiliations.

Copyright: © 2021 by the authors. Licensee MDPI, Basel, Switzerland. This article is an open access article distributed under the terms and conditions of the Creative Commons Attribution (CC BY) license (http://creativecommons.org/licenses/by/4.0/).

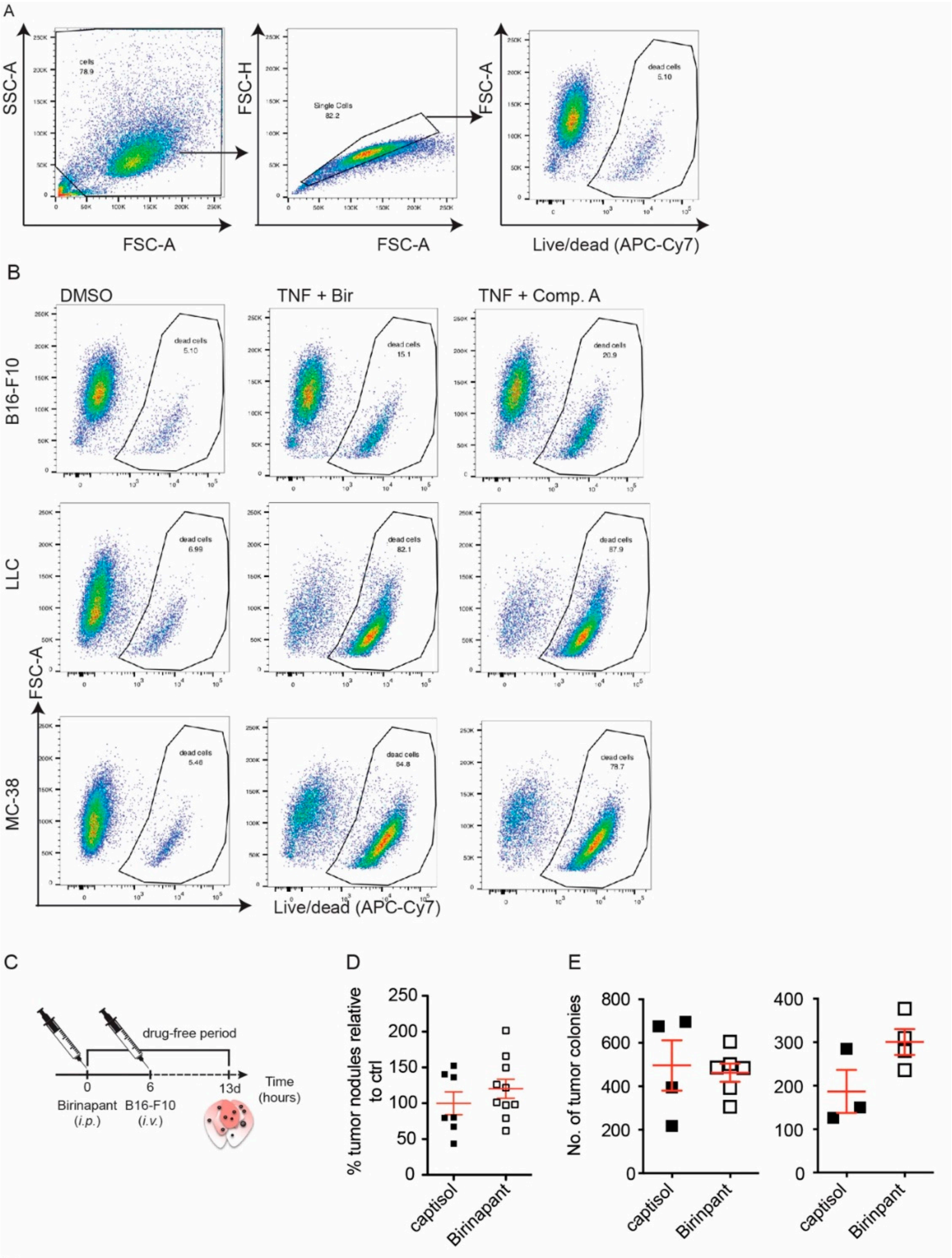

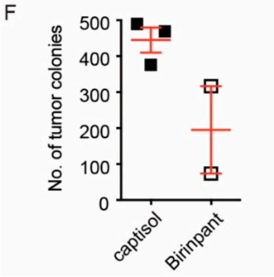

Figure S1. Cell viability analysis of different cancer cell lines upon Smac mimetics treatment.
(a) Gating strategy for live/dead stainig with eFLUOR780. **(b)** Representative flow cytometry scatter blots of the cell viability of different cancer cell lines upon Smac mimetics and TNF treatment. **(c)** Schematic for delivery of birinapant or vehicle (captisol) administered 6 hours prior to injection of tumor cells. **(d)** Percentage of superficial B16-F10 tumor nodules normalized to the average number of tumor nodules counted in the vehicle control in each independent experiment. **(e)** Primary data for tumor number in response to birinapant injection. **(f)** Additional primary data for tumor number in response to birinapant injection at 72h and 24h prior to tumor cell injection by tail vein iv. Each data point represents data obtained from one animal. Data are presented as mean ± SEM.

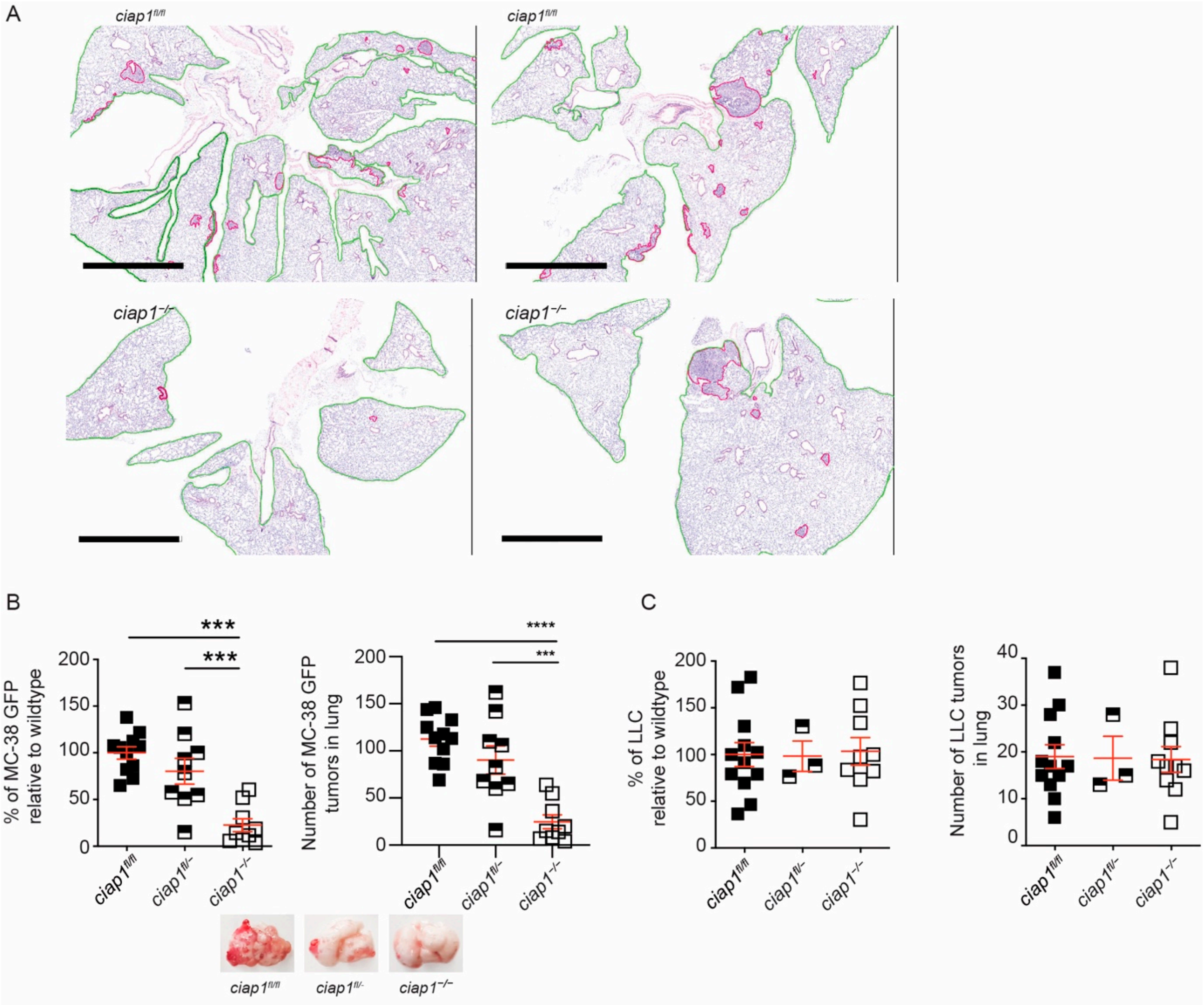

Figure S2. Tumor cells are preferentially reduced in lungs of cIAP1$^{-/-}$ mice but not due to immune rejection. **(a)** Representative H&E histology of lungs from *ciap1$^{fl/fl}$* and *ciap1$^{-/-}$* post 13 days injection of B16-F10 after segmentation. Lung tissue is outlined in green, tumor tissue is outlined in pink. **(b)** MC-38 GFP tumor cells were injected *i.v.* and nodules formed in the lung were counted 21 days later. Percentage of superficial MC-38 GFP tumor nodules normalized to the average of the wt control. Primary tumor nodule counts are also shown. **(d)** Percentage of superficial LLC tumor nodules normalized to the average of the wt control. Tumor cells were injected *i.v.* and nodules formed in the lung were counted 21 days later. Primary tumor nodule counts are also shown. Each data point represents an individual mouse. One way ANOVA with Bonferroni's test.(c). Data are presented as mean ± SEM. *p < 0.05; **p < 0.01; ***p < 0.001, ns = not significant.

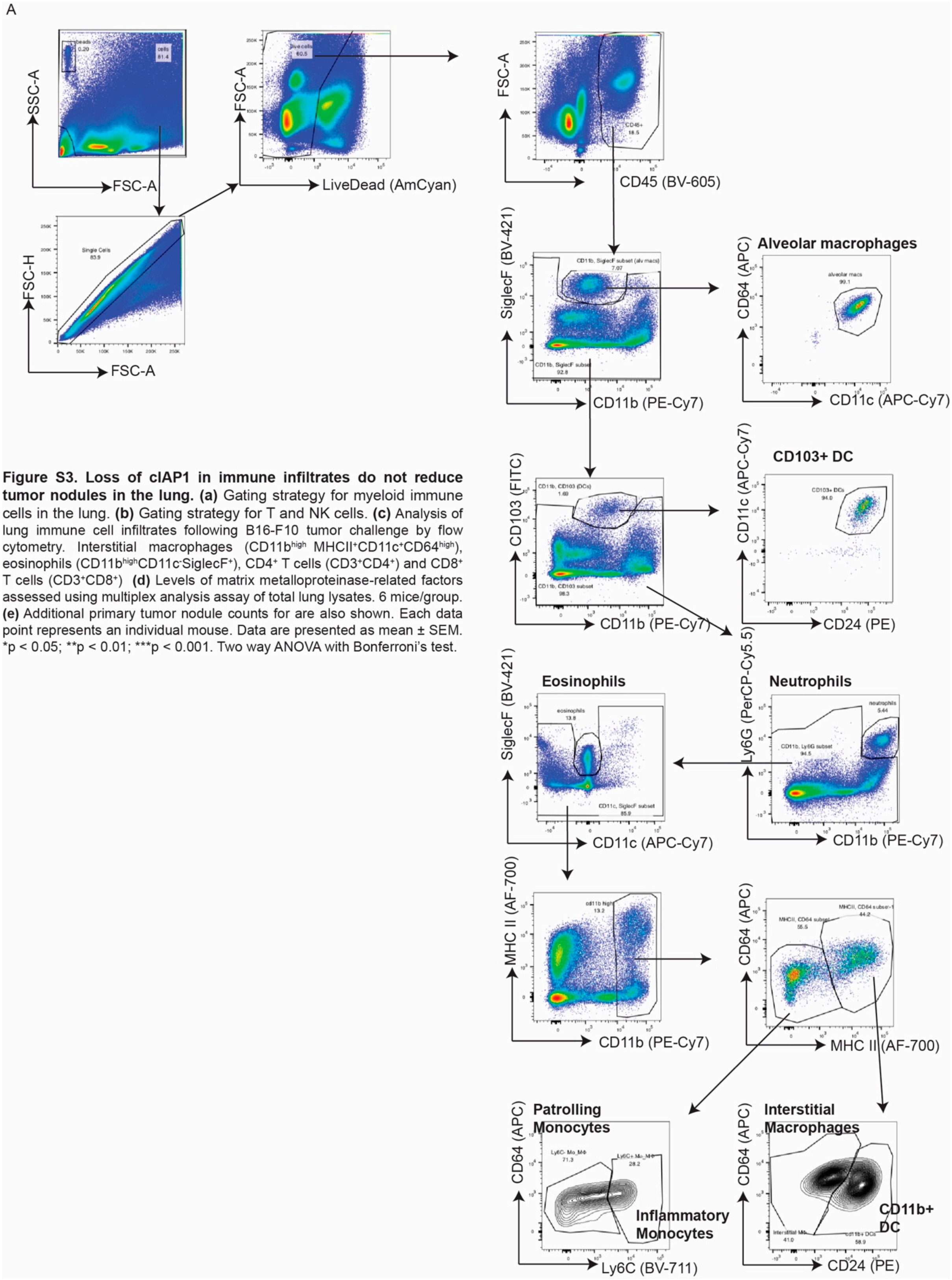

Figure S3. Loss of cIAP1 in immune infiltrates do not reduce tumor nodules in the lung. (a) Gating strategy for myeloid immune cells in the lung. **(b)** Gating strategy for T and NK cells. **(c)** Analysis of lung immune cell infiltrates following B16-F10 tumor challenge by flow cytometry. Interstitial macrophages ($CD11b^{high}$ $MHCII^{+}CD11c^{+}CD64^{high}$), eosinophils ($CD11b^{high}CD11c^{-}SiglecF^{+}$), $CD4^{+}$ T cells ($CD3^{+}CD4^{+}$) and $CD8^{+}$ T cells ($CD3^{+}CD8^{+}$) **(d)** Levels of matrix metalloproteinase-related factors assessed using multiplex analysis assay of total lung lysates. 6 mice/group. **(e)** Additional primary tumor nodule counts for are also shown. Each data point represents an individual mouse. Data are presented as mean ± SEM. *p < 0.05; **p < 0.01; ***p < 0.001. Two way ANOVA with Bonferroni's test.

Figure S3 (cont).

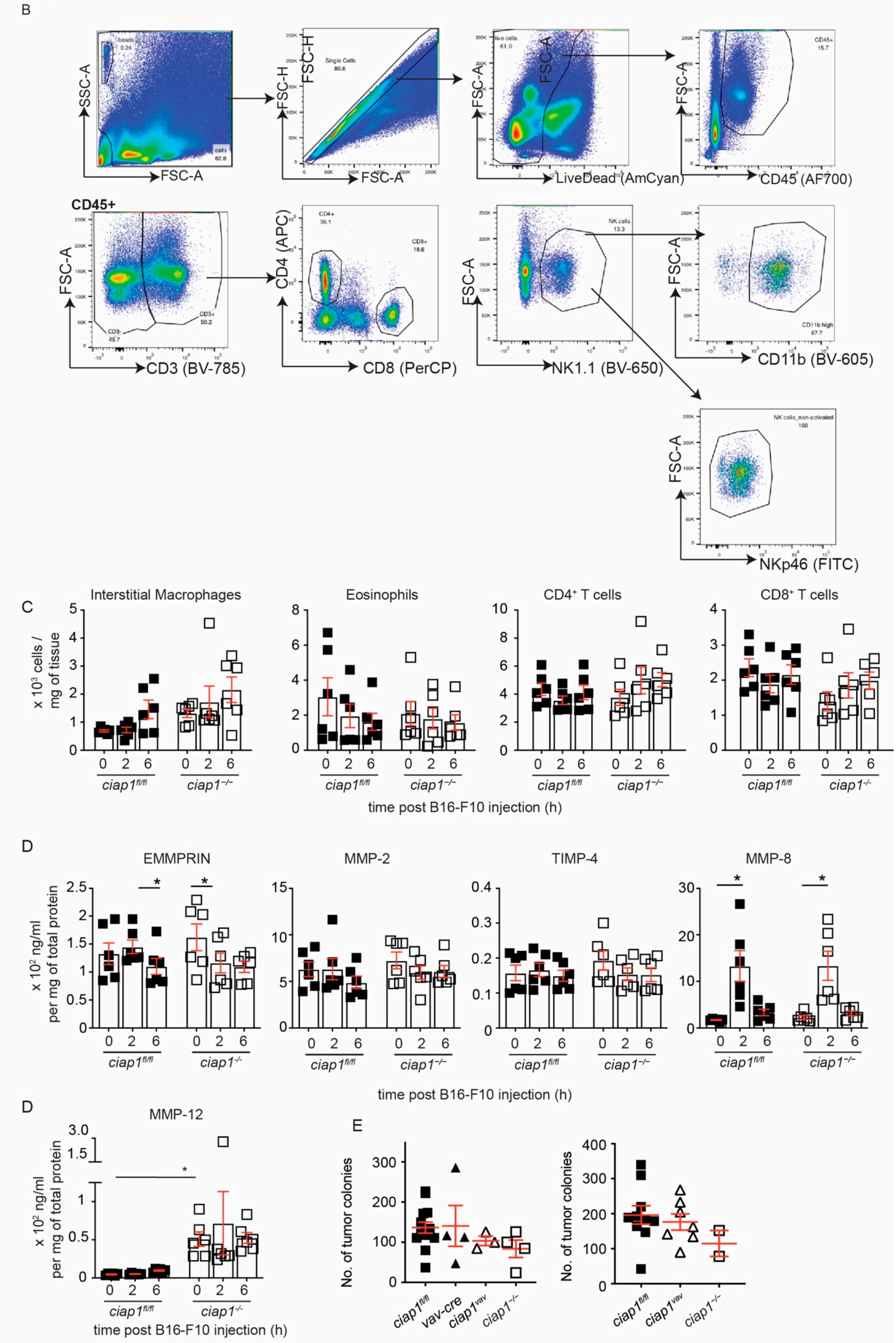

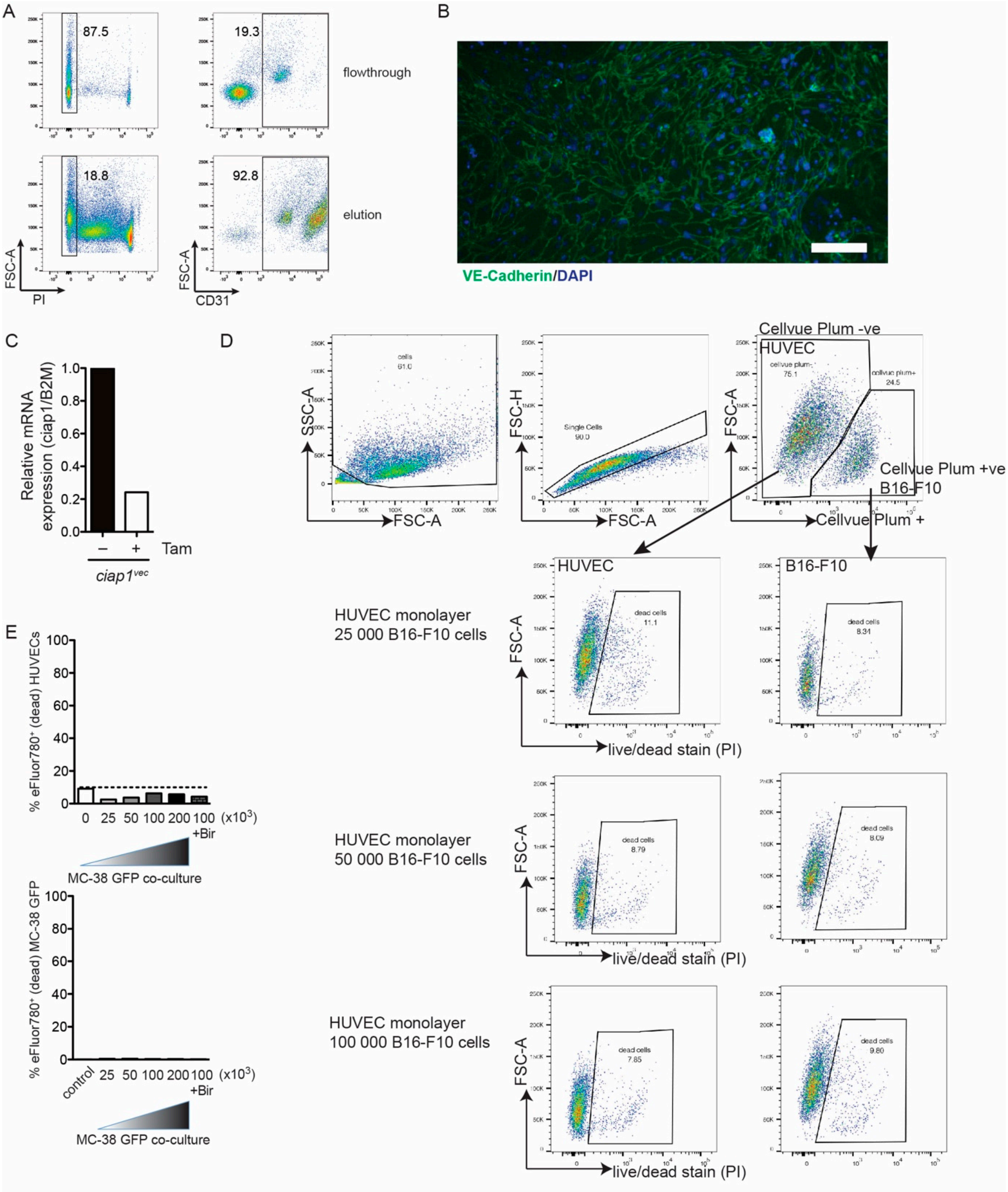

Figure S4. Isolation of primary endothelial cells (ECs), verification of cIAP1 loss by tamoxifen driven cre and analysis of cell death of MC-38 cells co-cultured with HUVEC monolayers.
(a) Primary isolated pulmonary ECs (CD31+) were analysed by flow cytometry following MACS separation. Cells from the elution, enriched in CD31+ cells, and flowthrough were stained with an anti-rat-FITC secondary antibody and propidium iodide (PI) to assay for live cells. Pre-gated on singlets. Representative FACS plots. **(b)** Immunofluorescence staining of primary isolated ECs with DAPI (blue) and primary antibody against VE-Cadherin (green). Representative picture. Scale bar: 100 µm. **(c)** Relative quantification of *ciap1* expression levels by qPCR. Total RNA was isolated from lung primary endothelial cells of untreated or tamoxifen-fed (oral gavage, tam) *ciap1vec* mice to induce recombination. **(d)** Gating strategy for co-culture of HUVEC and tumor cells using a membrane dye (CellVue Plum) and fixable live/dead stain (Amcyan). Representative facs plots are shown for the co-culture with increasing ratio of HUVEC:tumor cells. **(e)** HUVEC monolayers were co-cultured with increasing numbers of MC-38 GFP cells, with or without Birinapant, for 24h. Cell death of HUVECs and tumor cells was assessed by fixable viability eFluor780 dye uptake using flow cytometry. 3 independent experiments. Data are presented as mean ± SEM.

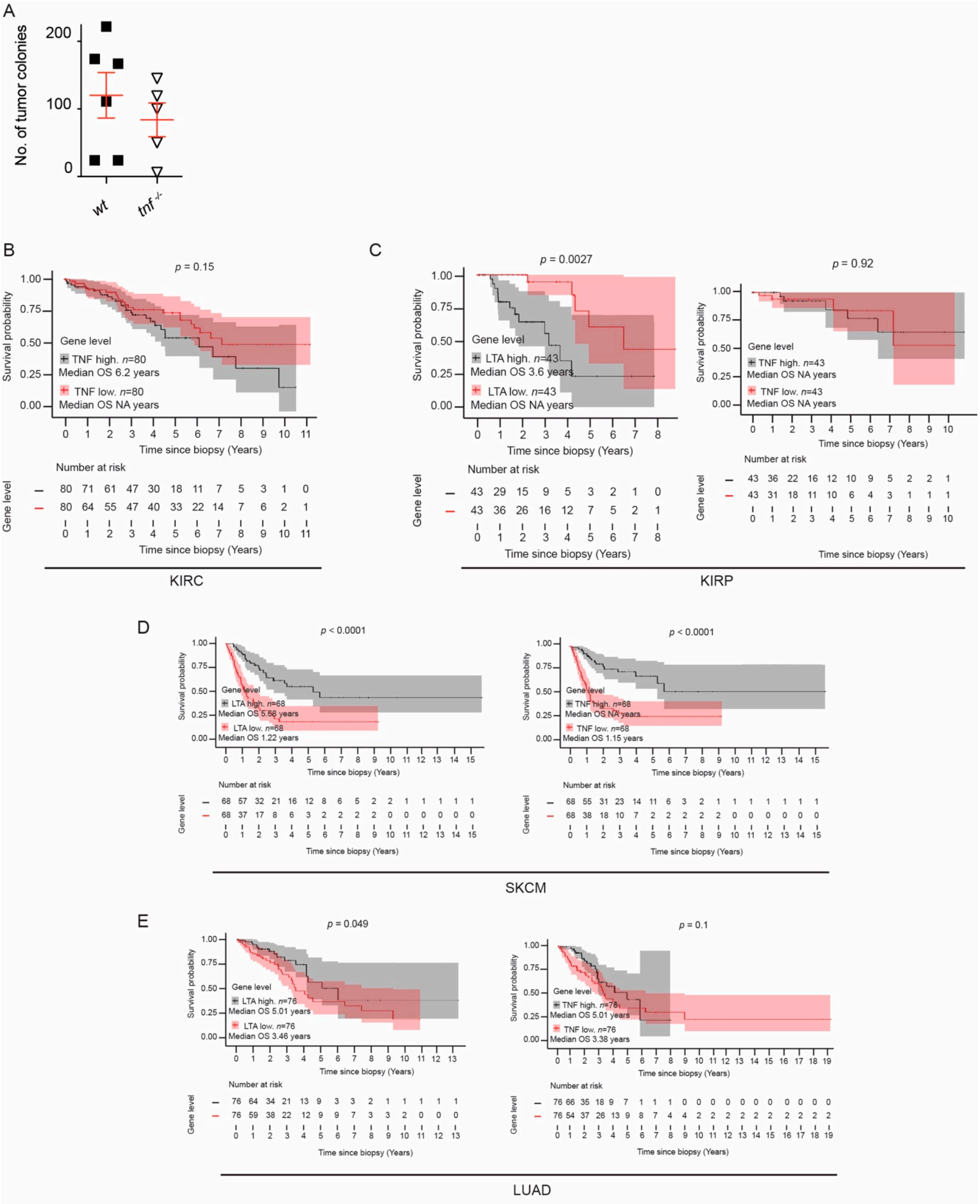

Figure S5. Correlation between survival of cancer patients and expression levels of LTA and TNF.
(a) Additional primary tumor nodule counts in wildtype and tnf-/- mice challenged with B16-F10 tumor cells. **(b-e)** Survival curves of high (>85th percentile) versus low (<15th percentile) LTA and TNF mRNA expression in kidney renal clear cell carcinoma (KIRC), kidney renal papillary cell carcinoma (KIRP), skin cutaneous melanoma (SKCM) and lung adenocarcinoma (LUAD) from TCGA data.

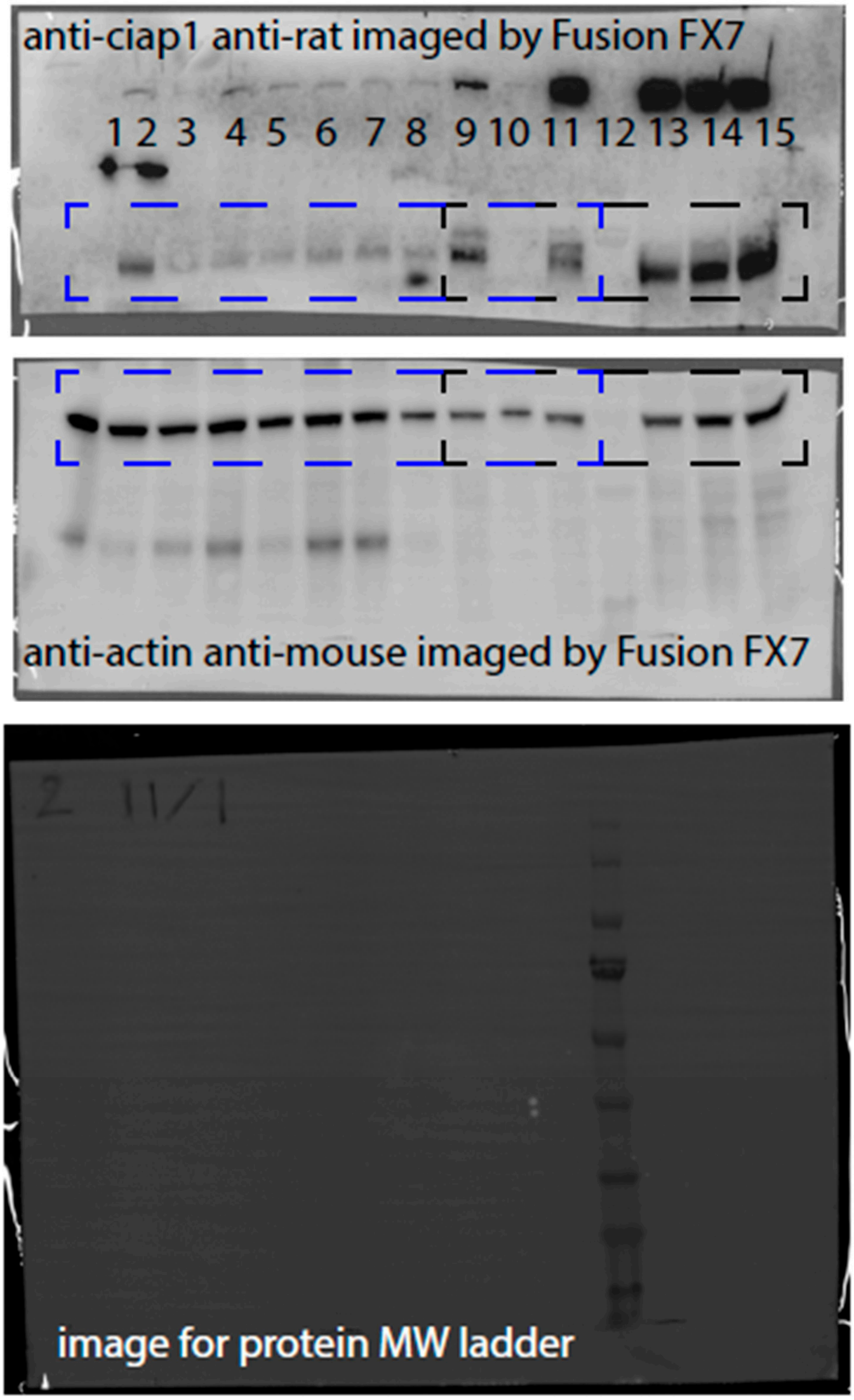
anti-ciap1 anti-rat imaged by Fusion FX7
1 2 3 4 5 6 7 8 9 10 11 12 13 14 15
anti-actin anti-mouse imaged by Fusion FX7
image for protein MW ladder

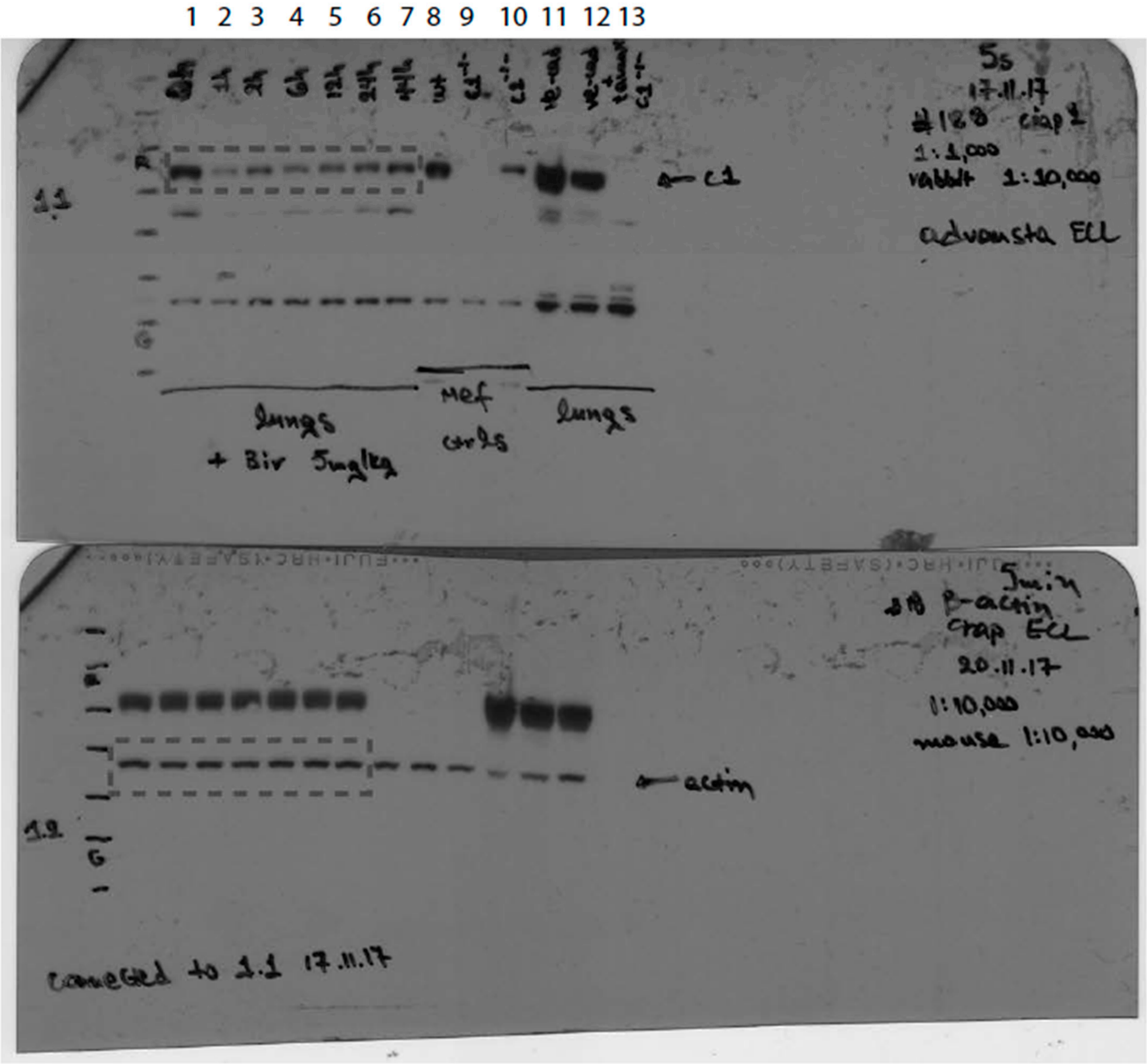
1 2 3 4 5 6 7 8 9 10 11 12 13
C1
lungs
+ Biv 5mg/kg
mef cells
lungs
17.11.17
1:1,000
rabbit 1:10,000
advansta ECL
actin
β-actin
20.11.17
1:10,000
mouse 1:10,000

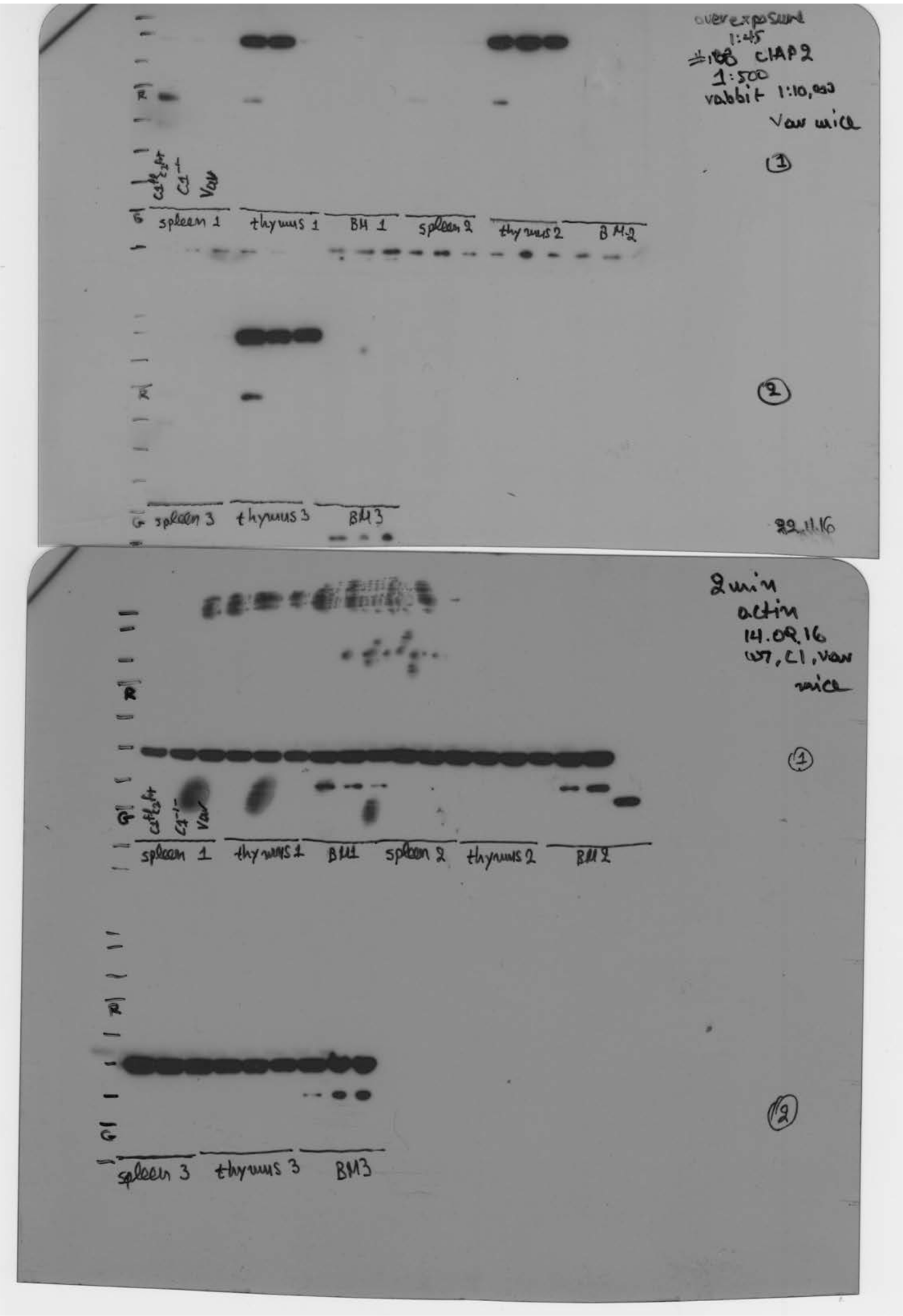

Figure S6. The uncropped Western blots.
